# Circ_0047339 promotes the activation of fibroblasts and affects the development of urethral stricture by targeting the miR-4691-5p/TSP-1 axis

**DOI:** 10.1038/s41598-022-19141-4

**Published:** 2022-08-30

**Authors:** Ke Ding, Daoyuan Li, Rui Zhang, Meilin Zuo

**Affiliations:** 1 Department of Urology, Xiangya Hospital, Central South University, Changsha, China; 2 Department of Urology, Hainan Affiliated Hospital of Hainan Medical University, Haikou, China; 3 Department of Urology, Hainan General Hospital, Haikou, China; 4 Hunan Traditional Chinese Medical College, Changsha, China; 5 Department of Anesthesiology, Second Xiangya Hospital, Central South University, Changsha, 410011 Hunan China

**Keywords:** Cell biology, Physiology, Medical research, Urology

## Abstract

Urethral stricture is related to scar tissue fibrosis, but its pathogenesis is still unclear. This study aims to explore the regulatory mechanism of circular RNA (circRNA) in the occurrence and development of urethral stricture. CircRNA microarray was employed to analyze circRNA expression profiles between human urethral scar tissue and normal urethral tissue. The results of circRNA microarray showed that there were 296 differentially expressed genes between urethral scar tissue and normal urethral tissue. The enrichment analysis of Kyoto encyclopedia of genes and genomes showed that these circRNAs were significantly correlated with ECM–receptor interaction. The first nine differentially expressed circRNA were selected to predict the circRNA–miRNA network. RT-qPCR results showed that circ_0047339 was upregulated considerably in urethral scar tissue. Urethral scar fibroblasts were isolated from human urethral scar tissue and cultured in vitro. After silencing circ_0047339, the proliferation of urethral scar cells decreased significantly, and the expressions of Collagen I (COL-1) and α-smooth muscle actin (α-SMA) also reduced. As a competing endogenous RNA, circ_0047339 could increase the expression of TSP-1 by competitively binding miR-4691-5p. In addition, miR-4691-5p mimic transfection could inhibit the proliferation of urethral scar fibroblasts and the presentation of thrombospondin-1 (TSP-1), α-SMA and COL-1, while circ_0047339 overexpression eliminated this inhibition. Our results showed that circ_0047339 might promote the growth and fibrosis of urethral scar fibroblasts through miR-4691-5p/TSP-1 axis, thus promoting the development of urethral stricture.

## Introduction

Urethral stricture is defined as the narrowing of the urethra related to fibrosis scar formation, a relatively common disease in urology practice. Its incidence rate in susceptible populations is estimated to be 0.6%, who are typically aged men^1–3^. It will lead to obstructive and irritating urinary symptoms, which affect the whole urinary tract, ultimately impair kidney function, and finally seriously reduce patients' quality of life^4^. With the development of modern medicine and surgery, various surgical options can be used to treat urethral stricture, including urethral dilatation, urethrotomy, and urethroplasty^5,6^. However, the current surgical treatment shows a high failure rate due to the new epithelial damage and the elimination of concentric fibrous scars in the lumen^7^. Among them, the failure rate of urethral dilatation and urethrotomy is as high as 90%^8,9^. The long-term effect is poor^8,9^, and 25% of urethroplasty recurs after 6 months^10^. Therefore, it is an urgent problem to discover a new treatment method for urethral stricture.

The pathophysiology of urethral stricture is mostly unknown. Fibrosis of urethral mucosa and surrounding corpus cavernosum after infectious, inflammatory or traumatic injury may lead to stenosis^11,12^. Pathogenesis includes fibroblast proliferation, collagen synthesis and extracellular matrix (ECM) deposition^13^.

Thrombospondin-1 (TSP-1) is an ECM glycoprotein, which can mediate cell–matrix and cell–cell interactions^14^. TSP-1 is an endogenous activator of transforming growth factor-beta (TGF-β), which is involved in developing many fibrosis diseases, including liver fibrosis, renal fibrosis and fibrosis complications of multiple myeloma^15,16^. Eliminating TSP-1 function in renal fibrosis induced by unilateral ureteral obstruction (UUO) can prevent interstitial fibrosis^17^. However, it is unclear whether TSP-1 is involved in developing fibrosis in urethral stricture.

Gene therapy has become a promising treatment option for many diseases^18^. Circular RNA (circRNA) is a new endogenous non-coding RNA that has attracted research interest^19^. Previous studies show that circRNA is involved in many disease processes, including renal and liver fibrosis^20,21^. In the reports of fibrosis-related diseases, like myocardial fibrosis^22^, diabetic nephropathy^23^ and pulmonary fibrosis^24^, it has been revealed that circRNA acts as a molecular sponge, which can isolate miRNA molecules and prevent the mechanism of targeting mRNA. However, there is little research on the role of circRNA in urethral fibrosis.

Therefore, this study aims to investigate circRNA imbalance in urethral fibrosis to broaden the understanding of the underlying pathological mechanism and find new therapeutic targets. Through circRNA sequencing, temporary circRNA expression profiles were generated in the urethral scar and normal urethral tissue. Differentially expressed circRNAs were obtained. CircRNA–miRNA targeting TSP-1 was selected for interaction. Through cell experiments, the mechanism of circRNA–miRNA targeting TSP-1 in urethral fibrosis was clarified to provide a new target and theoretical basis for treating urethral fibrosis-related diseases.

## Materials and methods

### Source of clinical specimens

From Jan. 2021 to Jun. 2021, six pairs of urethral scar and normal urethral tissue were collected in Xiangya Hospital. Patients' baseline information was summarized in Table 1. No matter it is trauma or iatrogenic stenosis, all patients with urethral stenosis underwent surgery at 2 months after the onset of the cause. The surgical specimens were free of local infection and ulcer, and no drug or radiation therapy was given before surgical resection. All specimens were obtained with the consent of patients and their families before operation and confirmed by pathological examination. The procedure used in this research followed the tenets of the Declaration of Helsinki and was approved by the Medical Ethics Committee of Xiangya Hospital Central South University.

**Table 1:** Patient demographics in this study.

Number of subject	Age	Gender	Etiology
1	61	Male	Trauma
2	56	Male	Trauma
3	71	Male	Iatrogenic
4	43	Male	Trauma
5	48	Male	Trauma
6	52	Male	Trauma

### CircRNA microarray analysis

The circRNA expression profile of the urethral scar and normal urethral tissue was detected using CapitalBio Technology Human CircRNA Array v2 (CapitalBio Technology, China). The total RNA of three urethral scar tissue and three normal urethral tissue were extracted. The purity and concentration of the RNA were determined by a NanoDrop ND-1000 instrument (Thermo Scientific, USA). The extracted RNAs were amplified and had reverse transcription into cDNA, then labeled with Cy3-dCTP. After purification, the labeled DNAs were hybridized into a microarray (CapitalBio Technology Human CircRNA Array v2). The circRNAs expression difference and statistical significance P value were calculated by GeneSpring GX software. Cluster analysis and graphical display were performed with Cluster3.0 software. The differential comparison is conducted to obtain differential genes according to the grouping information. Kyoto encyclopedia of genes and genomes (KEGG) Pathway analysis was performed on the linear mRNA transcripts corresponding to different circRNA^25^. The miRNA that circRNA might bind to was predicted by bioinformatics.

### Primary cell extraction and culture

The urethral scar tissue or normal urethral tissue was repeatedly rinsed in PBS containing antibiotics to remove redundant epithelial tissue. The tissues were divided into small pieces of 5 mm × 5 mm in size. Then, we dropped the culture medium onto the tissue to keep it moist. The tissues were put into a centrifuge tube. We added 0.25% trypsin digestive juice-EDTA and collagenase, digested it in a constant temperature water bath at 37 °C for 4–5 h, and shake it once every hour. We filtered the suspension with a filter screen, took the filtered suspension for centrifugation, discard the supernatant, added 6 mL of fresh culture solution, and cultured it in a cell incubator at 37 °C and 5% CO_2_. The next day, most of the fibroblasts adhered to the wall, the cell culture medium was changed, and the cells were changed every 2–3 days. Finally, urethral scar fibroblasts and normal urethral scar fibroblasts were successfully separated and cultured. The α-smooth muscle actin (α-SMA) was selected as a marker of fibroblasts for detection.

### Cell transfection

The small interference RNA (siRNA) specifically targeting circ_0047339 (si-circ_0047339), Lentivirus harboring circ_0047339 (LV-circ_0047339), miR-4691-5p mimic, and their corresponding negative controls (si-NC, vector, mimic NC) were obtained from honorgene Company (Changsha, China). According to the manufacturer's protocol, cells were transfected with Lipofectamine 3000 reagent (ThermoFisher, USA)^26^.

### Western blot

Total protein was extracted from the collected cells by RIPA lysate (AWB0136, Abiowell) containing protease inhibitor (583794, Jintai Hongda, Beijing, China) and protein phosphatase inhibitor (AWH0650, Abiowell). Then, the protein was transferred to the nitrocellulose (NC) membrane after 10% SDS-PAGE treatment. The membrane was sealed with 5% skim milk (AWB0004, Abiowell) at room temperature for 2 h. Collagen I (COL-1, 1:10,000, 67288-1-Ig, proteintech), α-SMA (1:6000, 14395-1-AP, proteintech), TSP-1 (1:1000, 18304-1-AP, proteintech), β-actin (1:5000, 66009-1-Ig, proteintech) were incubated with the membrane overnight at 4 °C. Then, the corresponding secondary antibodies HRP goat anti-mouse IgG (1:5000, SA00001-1, proteintech) or HRP goat anti-rabbit IgG (1:6000, SA00001-2, proteintech) were incubated with the membrane at room temperature for 2 h. The membrane was incubated with SuperECL Plus (AWB0005, abiowell). Then, the protein bands were visualized by a chemiluminescence imaging system (Chemiscope 6100, Clinx, China). The relative content of protein is expressed as target protein/β-actin (Supplementary Figs. S1–S4).

### Quantitative reverse transcription PCR (RT-qPCR)

Trizol kit (15596026, Thermo) was adopted to extract the total RNA of cells. RNA was reversely transcribed into cDNA by reverse transcription kit (CW2569, CW2141, Beijing Kangwei Century). RT-qPCR was carried out under the following reaction conditions using UltraSYBR Mixture (CW2601, Beijing Kangwei Century), cDNA and primers. The condition was 95 °C for 10 min, 95 °C for 15 s, 60 °C for 30 s, with 40 cycles. GAPDH is the internal control of genes, and U6 is the internal control of miRNAs. The formula is 2^−ΔΔCt^. The primers used in this study are Table 2.

**Table 2:** Primer sequences.

Gene	Sequences (5′–3′)
hsa_circ_0005413	Forward: GGACCTCTTTCAATGACAACGC
	Reverse: CCATCTGTTGCCAAACCACT
hsa_circ_0006912	Forward: AGCCTACTGCAAATCCAAACAC
	Reverse: CAGGTTTCTTGCCTCTTGGTT
hsa_circ_0019957	Forward: AAAACATGCCCCAGAGTCCT
	Reverse: ACACTTGCCGATCGACTCCC
hsa_circ_0021726	Forward: GGACCTCTTTCAATGACAACGC
	Reverse: CATCATCAATGCCTGATCCAGA
hsa_circ_0021731	Forward: TTCAACCCAATCTCACACCCC
	Reverse: GGTGCCATTTCTGTCTACATGC
hsa_circ_0047338	Forward: TTATCCCAGTTCCTGATGGCT
	Reverse: TCCCACTCCAGAGATTCGGTA
hsa_circ_0047339	Forward: CCATGAGAACAAGGCATTCCAC
	Reverse: CTCCCGGTCGACTATAGCTG
hsa_circ_0047343	Forward: TAACAGCCAATGGAGCCGAT
	Reverse: TGTTCTAGCGGAGACAACCAC
hsa_circ_0093740	Forward: CACTTATCAAGCTGCCATACCTG
	Reverse: GGTCCTCCAGCAGTCCCTT
TSP-1	Forward: AAACACTGAAGCACACGCAAC
	Reverse: GACAGCTCCTCCCTCATCCAC
hsa-let-7b-5p	Forward: ACAGCAGGCACAGACAGGCAGT
	Reverse: GCTGTCAACGATACGCTACGTAA
hsa-miR-4691-5p	Forward: ACAGCAGGCACAGACAGGCAGT
	Reverse: GCTGTCAACGATACGCTACGTAA
hsa-miR-550b-2-5p	Forward: ACAGCAGGCACAGACAGGCAGT
	Reverse: GCTGTCAACGATACGCTACGTAA
hsa-miR-766-5p	Forward: ACAGCAGGCACAGACAGGCAGT
	Reverse: GCTGTCAACGATACGCTACGTAA
GAPDH	Forward: ACAGCCTCAAGATCATCAGC
	Reverse: GGTCATGAGTCCTTCCACGAT
U6	Forward: CTCGCTTCGGCAGCACA
	Reverse: AACGCTTCACGAATTTGCGT

### Immunofluorescence (IF) analysis

COL-1 and α-SMA expressions were measured by IF analysis. The cell slides were fixed with 4% paraformaldehyde for 30 min. They were washed with PBS 3 times and blocked with 5% BSA at 37 °C for 60 min. The slides were incubated with the first antibody (COL-1 (1:50, 67288-1-Ig, proteintech), α-SMA (1:50, ab7817, abcam)) at 4 °C overnight. Then, the CoraLite594-conjugated Goat Anti-Mouse IgG (H + L) (1:200, SA00013-3, proteintech) antibody was incubated with the slides at 37 °C for 90 min. DAPI working solution was stained at 37 °C for 10 min. The results were observed under a microscope (BA210T, Motic).

### Cell counting kit-8 (CCK-8) assay

According to the manufacturer's instructions, the CCK-8 kit (NU679, DOJINDO) was used to detect cell viability. The cells were inoculated into a 96-well plate (1 × 10^4^/well) and attached to the wall in a 5% CO2 incubator at 37 °C for 24 h. According to the demand, the cells were divided into different groups. Each group was provided with 6 compound holes 24 h after taking out the 96-well plate. We observed it under a microscope, added CCK-8 reagent and incubated it for 2 h. A microplate analyzer was utilized to detect the absorbance (OD) value at 450 nm, and the results were recorded.

### 5-Ethynyl-2′-deoxyuridine (EDU) assay

According to the manufacturer's instructions, cell proliferation was performed by the EDU assay kit (Guangzhou RiboBio). The cells were inoculated into 96-well plates (1 × 10^4^/well) and incubated with 4% paraformaldehyde at room temperature for 30 min. Then, the cells were treated with 100 μL of 1 × Apollo^®^ staining reaction solution for 30 min. Next, 100 μL Hoechst 33342 reaction solution was added to each well and incubated for 30 min. Immediately after dyeing, we used a microscope (DSZ2000X, Beijing Cnmicro instrumentco., Ltd) to observe and take pictures.

### Dual-luciferase reporter assay

According to the manufacturer's instructions, circ_0047339 wide type (WT) or circ_0047339 mutant (MUT) or TSP-1 3′UTR WT or TSP-1 3′UTR MUT, and miR-4691-5p mimic or NC mimic were co-transfected in 293A cells (HonorGene, Changsha) with Lipofectamine 300 transfection reagent (Invitrogen). The cells were digested with trypsin at 37 °C, and centrifuged (4 °C, 1000 rpm, 5 min) and the supernatant was discarded. The luciferase activity was measured using a dual-luciferase assay kit (E1910, Promega, USA).

### Statistics

Statistical analyses were conducted using GraphPad Prism 9 software (GraphPad Software, Inc., USA). Each experiment was conducted at least three times. Statistical analysis among more than two groups and between two groups was performed using ANOVA and Student's *t* test. Data are presented as means ± standard deviations. P < 0.05 was considered statistically significant, P < 0.01 was considered a significant statistical difference, and P < 0.001 was considered an extremely significant statistical difference.

### Ethical approval

The procedure used in this research followed the tenets of the Declaration of Helsinki and was approved by the Medical Ethics Committee of Xiangya Hospital Central South University (202112614). All specimens were obtained with the informed consent of patients and their families before operation and confirmed by pathological examination.

## Results

### CircRNA expression profile analysis of urethral scar tissue

**Figure 1:**
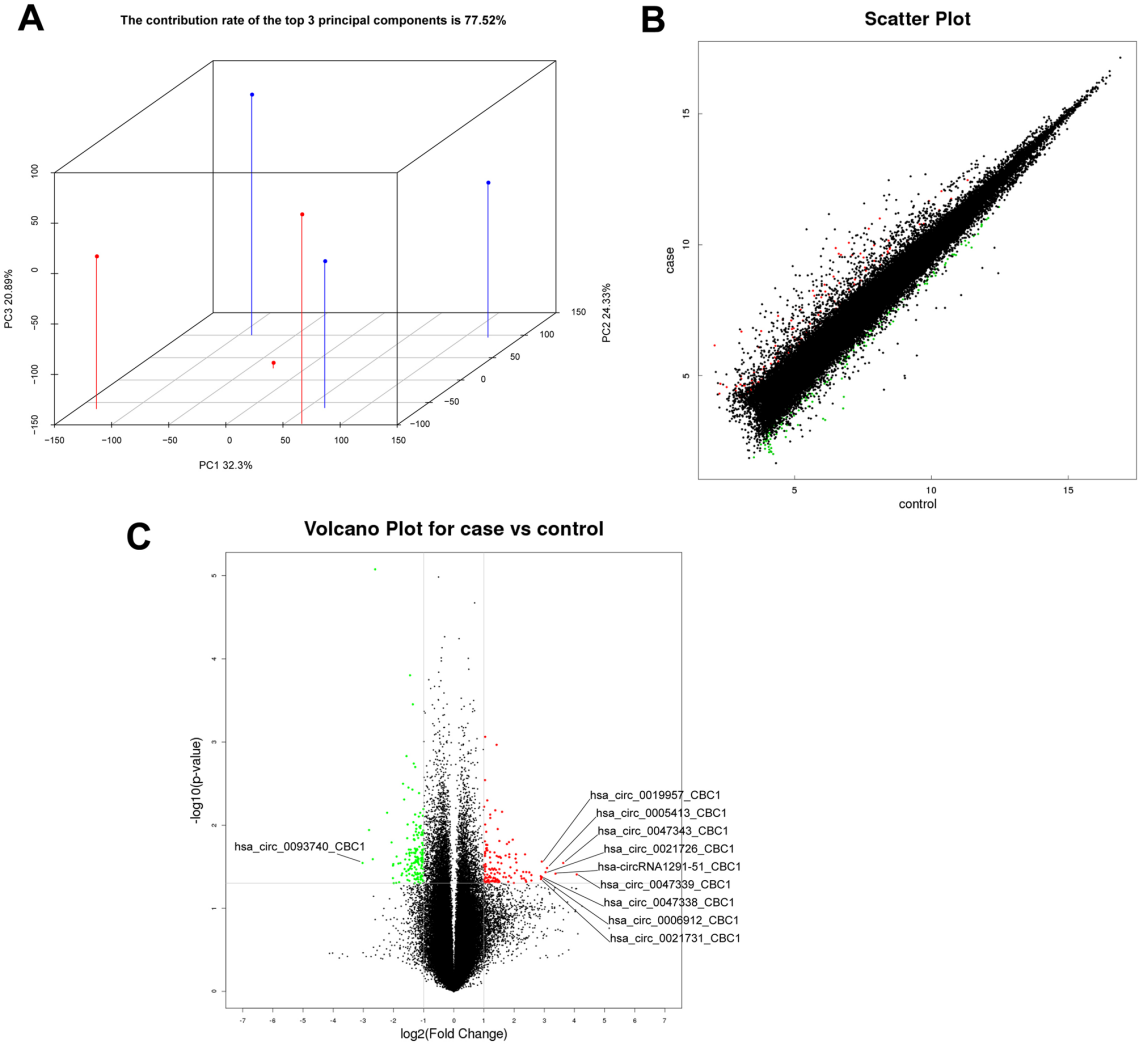
CircRNA expression profile analysis of urethral scar tissue. (**A**) Principal Component Analysis 3D diagram showed three principal components in three-dimensional space, PC1 was the first principal component, PC2 was the second principal component, and PC3 was the third principal component. The percentage in the coordinate axis was the contribution rate of each principal component. Duplicate samples of the same group were marked with the same shape and color. (**B**) Scatter chart showed the signal values of circRNA in each group of samples. (**C**) Volcano map showed the difference of circRNA in two groups of samples. Up-regulated circRNA was marked in red, down-regulated circRNA was marked in green, and circRNA with no significant difference was marked in black.

We performed circRNA microarray to identify the differential circRNA expression between human urethral scar tissue and normal urethral mucosa tissue. Then, we determined the characteristics of circRNA in the urethral scar. Principal Component Analysis (PCA) was a statistical method to reflect the similarity of samples. By dimensionality reduction of data, the expression of samples was displayed in three-dimensional space. The result showed that the contribution rate of the top 3 principal components is 77.52% (Fig. 1A). The scatter plot showed the difference in circRNA expression between samples in the urethral scar group (case) and the normal urethral mucosa tissue group (control). Among them, those marked in red were up-regulated circRNA, those marked in green were down-regulated circRNA, and those marked in black were circRNA with no significant difference (Fig. 1B). In addition, we drew a volcano map together with P-value and fold change (FC), which were obtained by difference analysis. Based on the threshold of FC ≥ 2 and P-value ≤ 0.05, 296 differential circRNA were identified (Fig. 1C). Among them, 166 circRNA expressions were down-regulated, and 130 circRNA expressions were upregulated in human urethral scar tissue, compared with normal urethral tissue.

### Interaction between circRNA and miRNA in urethral scar tissue

**Figure 2:**
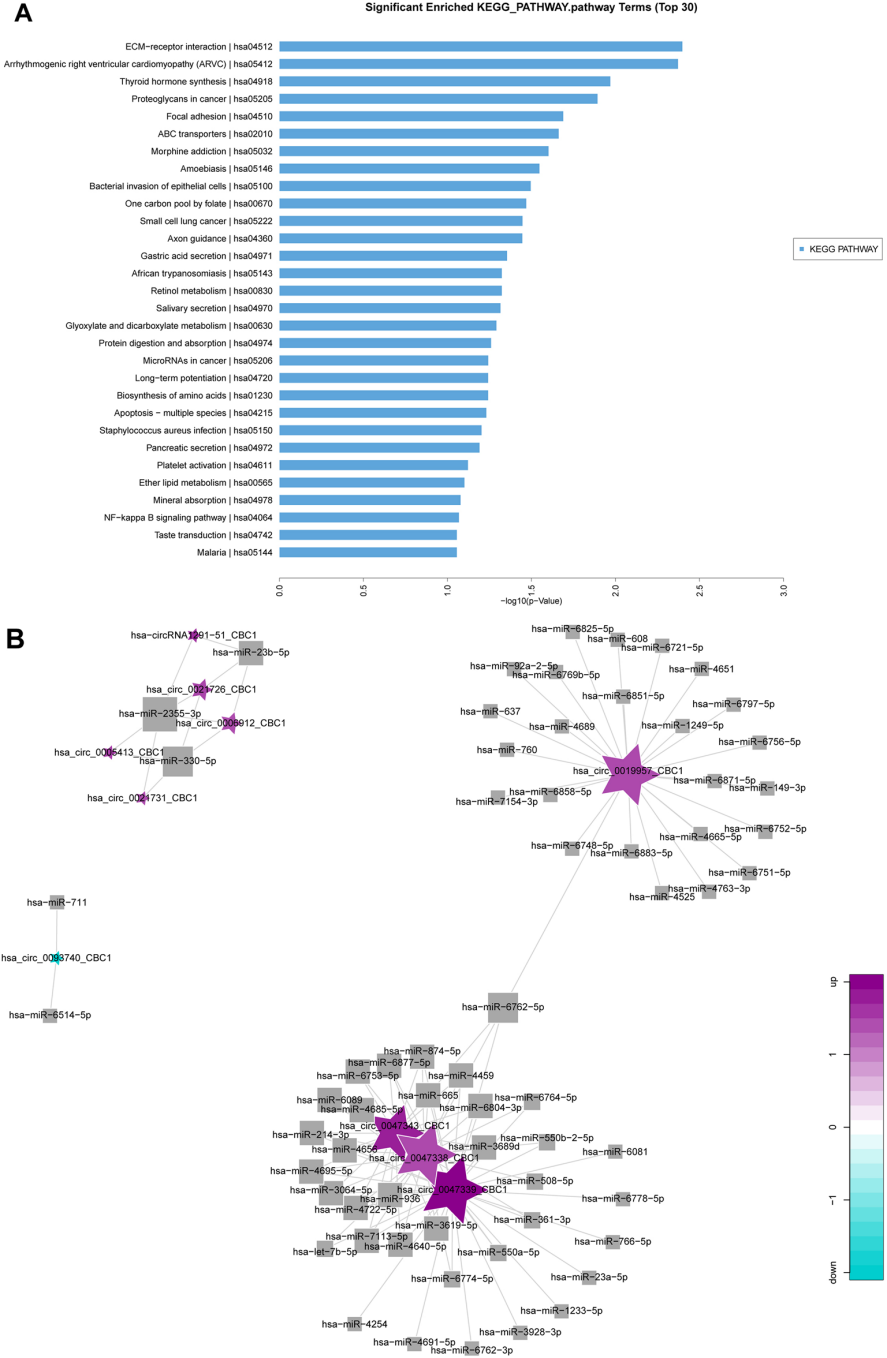
Interaction between circRNA and miRNA in urethral scar tissue. (**A**) KEGG showed the enrichment of differential circRNA functional pathways. (**B**) CircRNA–miRNA interaction analysis network diagram. Square represented miRNA, and pentagram represented circRNA, in which green was down-regulated and purple was upregulated. The size of a point meant the number of nodes connected to the point, and the larger the point, the more nodes connected to the point.

We next aimed to explore the functional changes caused by circRNA changes in patients with urethral fibrosis. Based on the microarray analysis data, the KEGG pathway of different circRNA groups was annotated and enriched. KEGG pathway statistical chart showed the first 30 enriched signal pathways (Fig. 2A). The differential circRNA was significantly related to ECM–receptor interaction, Arrhythmogenic right ventricular cardiomyopathy (ARVC), Thyroid hormone synthesis, and Proteoglycans in cancer and Focal adhesion. CircRNA could combine with miRNA in a targeted way and indirectly regulate the translation of mRNA^27^. The miRanda software was used to select the differentially expressed circRNA to predict the target miRNA, and the circRNA–miRNA network diagram was drawn. The results showed the interaction between the first ten most significantly expressed circRNA and its target miRNA (Fig. 2B).

### The expression of circ_0047339/miRNA/TSP-1 interaction in urethral scar tissue

**Figure 3:**
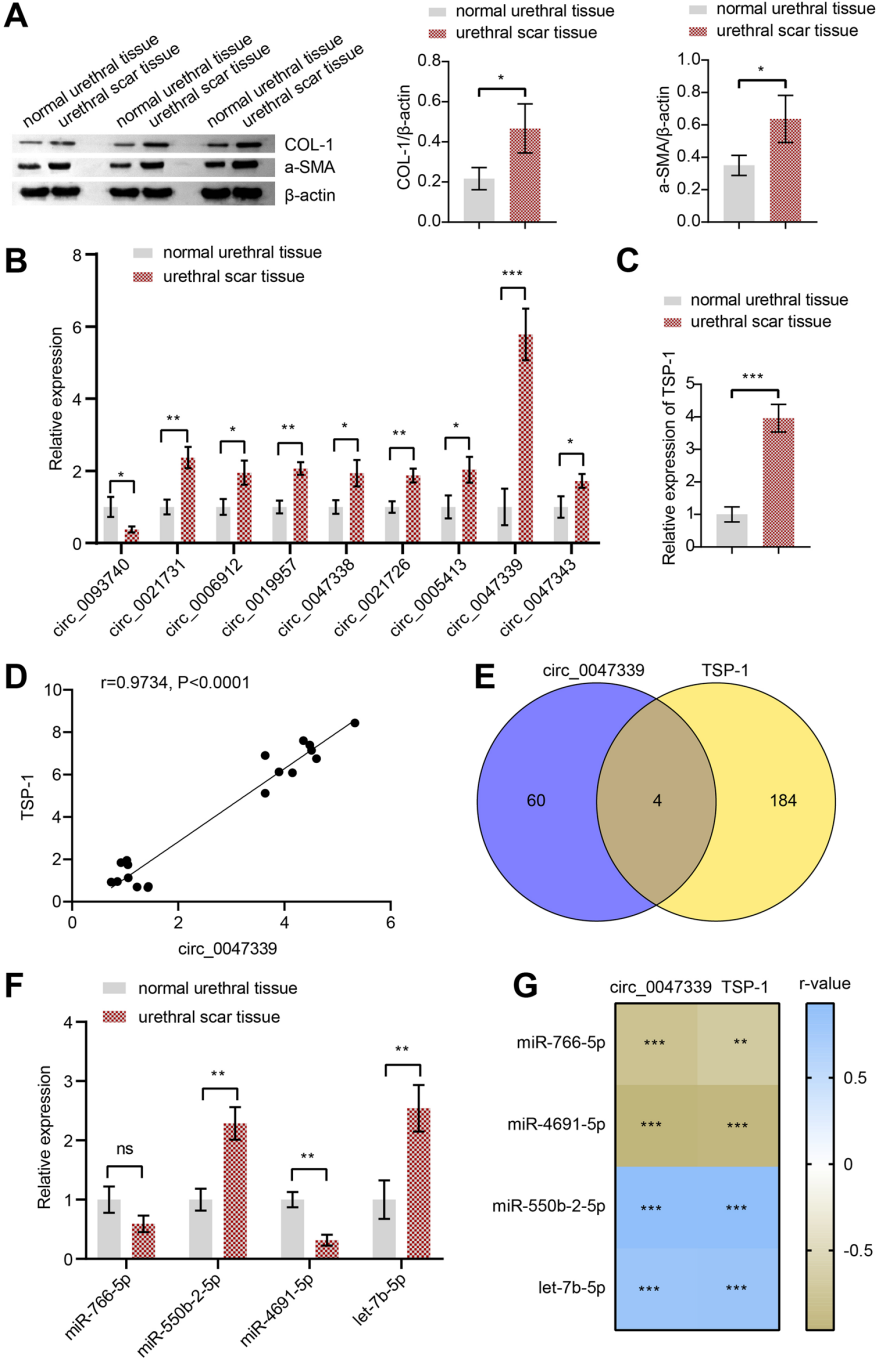
The expression of circ_0047339/miRNA/TSP-1 interaction in urethral scar tissue. (**A**) We performed western blot to verify the expression of COL-1 and α-SMA in urethral scar tissue. (**B**) We confirmed the expression of the first nine differential circRNA in the chip microarray analysis by RT-qPCR. (**C**) The expression of TSP-1 in urethral scar tissue was verified by RT-qPCR. (**D**) The correlation between TSP-1 and circ_0047339. (**E**) Venn diagram. (**F**) The expression of let-7b-5p, miR-4691-5p, miR-766-5p and miR-550b-2-5p were characterized by RT-qPCR in urethral scar tissue. (**G**) Correlation analysis of TSP-1 and circ_0047339 with let-7b-5p, miR-4691-5p, miR-766-5p and miR-550b-2-5p. Blue represents positive correlation, and brown represents negative correlation. A larger absolute value of the r-value indicates a stronger correlation. *P < 0.05, **P < 0.01, ***P < 0.001, *ns* no significance.

We collected human normal urethral and human urethral scar tissue, and determined the expressions of COL-1 and α-SMA via western blot assay. The expressions of COL-1 and α-SMA were significantly upregulated in human urethral scar tissue compared with normal urethral tissue (Fig. 3A). Next, we verified the expression of the first nine differential circRNA in the microarray analysis results by RT-qPCR. The results showed that circ_0047339 was upregulated in human urethral scar tissue, and the most significant difference (Fig. 3B). Therefore, we choose circ_0047339 for follow-up research. The above results showed that ECM–receptor interaction was the most significant KEGG pathway for differential circRNA enrichment. TSP-1 is a matricellular protein in the ECM, which mediates cell–matrix and cell–cell interactions^14,28^. We speculated that TSP-1 might be an important target gene downstream of circ_0047339. Therefore, the expression of TSP-1 was detected by RT-qPCR in urethral scar tissue. TSP-1 expression was upregulated in urethral scar tissue compared to normal urethral tissue (Fig. 3C). Correlation analysis helped us discovered that TSP-1 positively correlated with circ_0047339 (Fig. 3D). Next, we predicted the miRNAs targeted by circ_0047339 and the miRNAs targeted by TSP-1 by bioinformatics. The Venn diagram showed that circ_0047339 and TSP-1 co-targeted four miRNAs (Fig. 3E). The expression of four miRNAs in human urethral scar tissue was detected by RT-qPCR. The results revealed that miR-4691-5p in human urethral scar tissue was down-regulated, while miR-550b-2-5p and let-7b-5p were up-regulated (Fig. 3F). There was no significant difference in the expression of miR-766-5p between the two groups (Fig. 3F). Correlation analysis showed the expression of miR-766-5p and miR-4691-5p was significantly negatively correlated with circ_0047339 and TSP-1 (Fig. 3G). While the expression of miR-550b-2-5p and let-7b-5p was significantly positively correlated with circ_0047339 and TSP-1 (Fig. 3G). The above results indicated that there might be a sponge mechanism of circ_0047339/miR-4691-5p/TSP-1 in human urethral scar tissue.

### Circ_0047339 regulated the fibrosis of urethral fibroblasts

**Figure 4:**
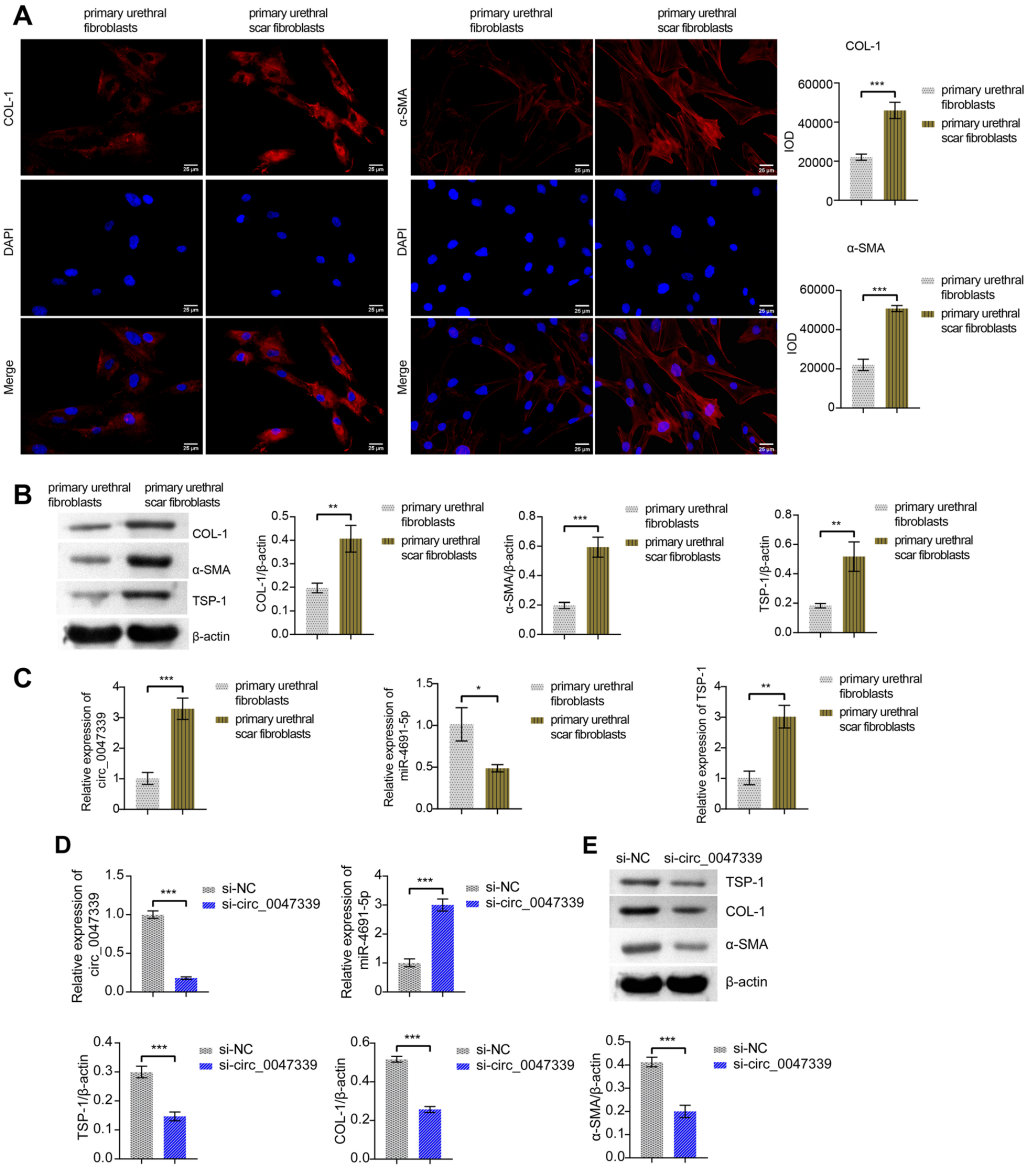

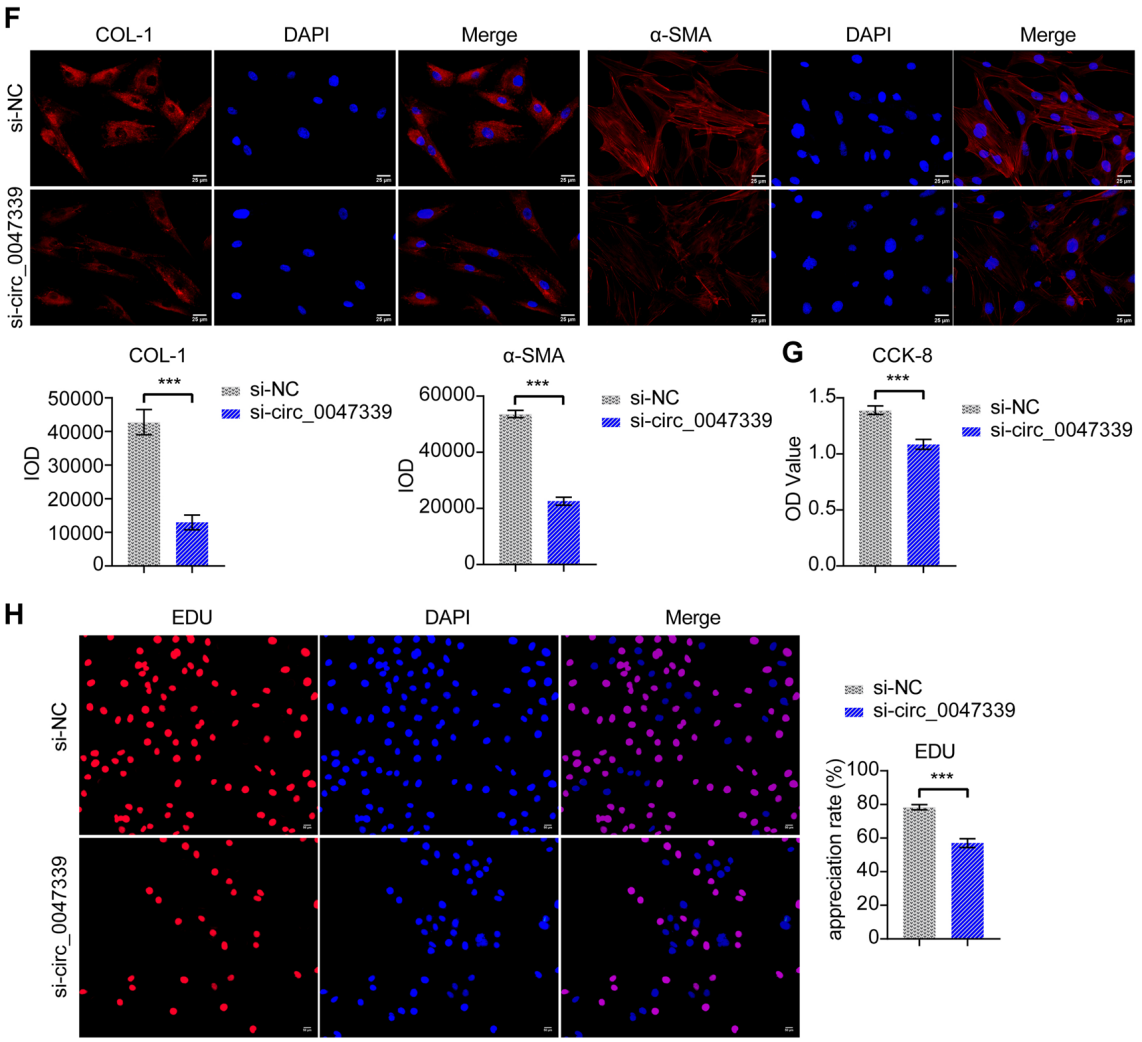
Circ_0047339 regulated the fibrosis of urethral fibroblasts. (**A**) The expressions of COL-1 and α-SMA were detected by IF, scale bar: 25 μm. (**B**) Western blot was conducted to determine the protein expression of COL-1, α-SMA, and TSP-1. (**C**) RT-qPCR was used to detect the expression of circ_0047339, miR-4691-5p and TSP-1. (**D**) The expression of circ_0047339 and miR-4691-5p was investigated by RT-qPCR. (**E**) Western blot was conducted to determine the protein expression of COL-1, α-SMA and TSP-1. (**F**) The expression of COL-1 and α-SMA was assessed by IF, scale bar: 25 μm. (**G**) CCK-8 was used to detect the cell viability. (**H**) EDU assay was utilized to detect cell proliferation, scale bar: 50 μm. *P < 0.05, **P < 0.01, ***P < 0.001.

Next, to study the function of circ_0047339, we extracted human primary urethral fibroblasts and human primary urethral scar fibroblasts. IF detection showed that the expression of COL-1 and α-SMA in human primary urethral scar fibroblasts was higher than that in human primary urethral fibroblasts (Fig. 4A). Western blot results were consistent with the IF results (Fig. 4B). In addition, the protein level of TSP-1 increased in human primary urethral scar fibroblasts (Fig. 4B). The results of RT-qPCR showed that circ_0047339 and TSP-1 were upregulated in human primary urethral scar fibroblasts, while miR-4691-5p was the opposite (Fig. 4C). The expression of circ_0047339 in human primary urethral scar fibroblasts was silenced by transfection of si-circ_0047339 (Fig. 4D). Meanwhile, the expression of miR-4691-5p in the si-circ_0047339 group was upregulated than in the si-NC group (Fig. 4D). Western blot results showed that the protein levels of TSP-1, COL-1 and α-SMA were down-regulated in si-circ_0047339 group compared to si-NC group (Fig. 4E). The results of IF were consistent with those of western blot (Fig. 4F). Compared with the si-NC group, the cell viability of si-circ_0047339 group decreased (Fig. 4G). The EDU test results also showed cell proliferation in the si-circ_0047339 group decreased (Fig. 4H). These results suggested that silencing si-circ_0047339 could reduce the vitality of urethral fibroblasts, inhibit proliferation and alleviate fibrosis.

### Circ_0047339 acts as a ceRNA in TSP-1 regulation by sponging miR-4691-5p

**Figure 5:**
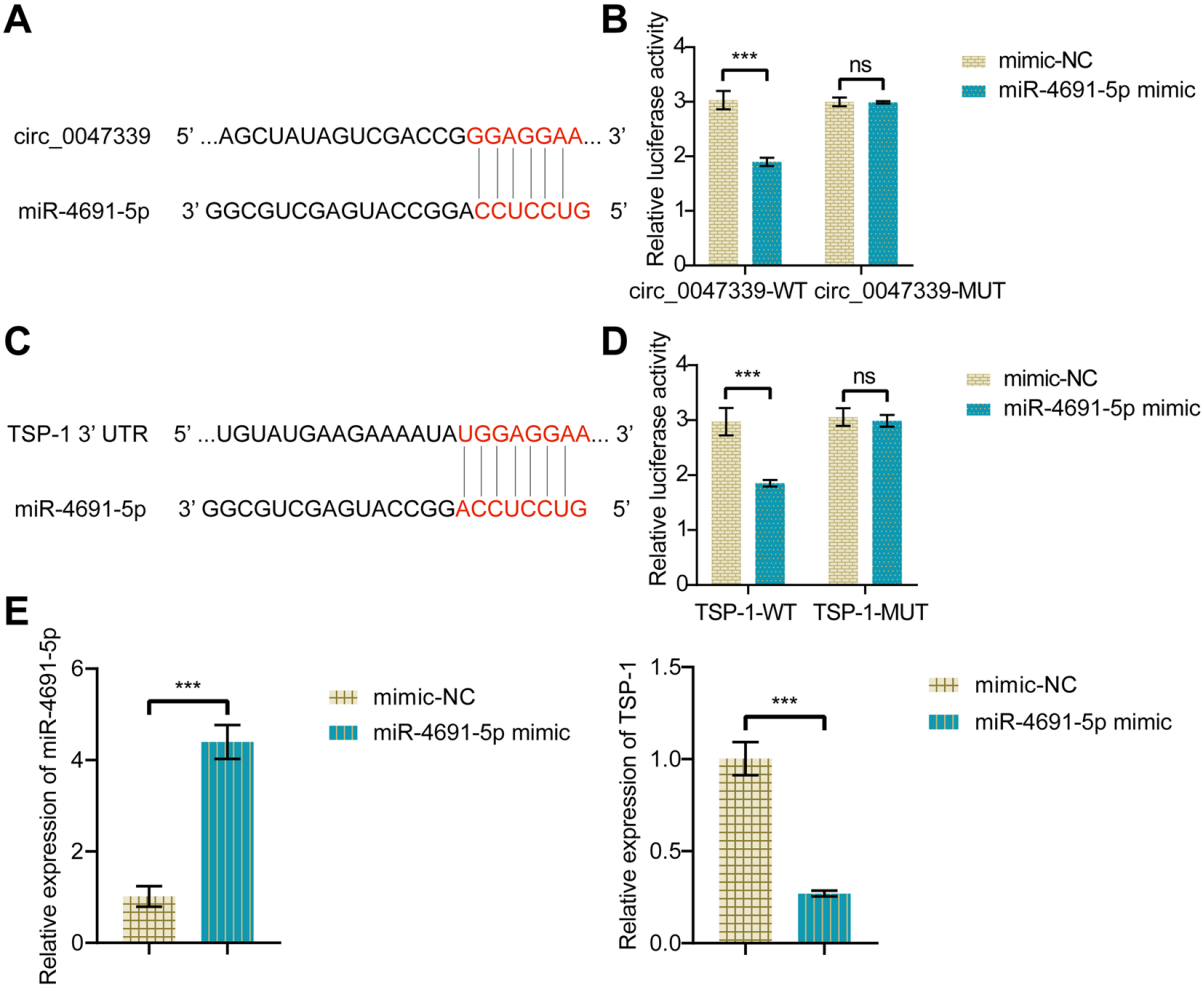
Circ_0047339 acts as a ceRNA in TSP-1 regulation by sponging miR-4691-5p. (**A**) The binding site of circ_0047339 and miR-4691-5p. (**B**) Dual-luciferase assay revealed the direct interaction of circ_0047339 with miR-4691-5p. (**C**) The binding site of miR-4691-5p and TSP-1. (**D**) Dual-luciferase assay revealed the binding of miR-4691-5p and TSP-1 3′UTR. (**E**) RT-qPCR was used to detect the expression of miR-4691-5p and TSP-1. *P < 0.05, **P < 0.01, ***P < 0.001. *ns* no significance.

The targeting relationship between circ_0047339 and miR-4691-5p was analyzed by bioinformatics. Figure 5A showed the targeted binding sites of circ_0047339 and miR-4691-5p. Dual-luciferase assay results showed that miR-4691-5p mimic transfection significantly reduced the luciferase activity of circ_0047339-WT, but did not reduce the luciferase activity of circ_0047339-MUT group (Fig. 5B). TSP-1 3′UTR contained the potential binding site of miR-4691-5p (Fig. 5C). Then the luciferase reporter plasmid was constructed, which includes 3′UTR regions of TSP-1 mRNA for luciferase detection. The results showed that miR-4691-5p mimic significantly inhibited the luciferase activity of TSP-1-WT, but had no effect on the TSP-1-MUT group (Fig. 5D). The expression level of TSP-1 mRNA in human primary urethral scar fibroblasts transfected with miR-4691-5p mimic was detected by RT-qPCR. The results showed that TSP-1 mRNA in the miR-4691-5p mimic group was significantly down-regulated compared with the mimic-NC group (Fig. 5E). These results indicated that circ_0047339 could be used as a molecular sponge of miR-4691-5p to regulate the expression of TSP-1.

### Circ_0047339/miR-4691-5p/TSP-1 interaction network was involved in regulating the proliferation, ECM deposition and collagen synthesis of urethral scar fibroblasts

**Figure 6:**
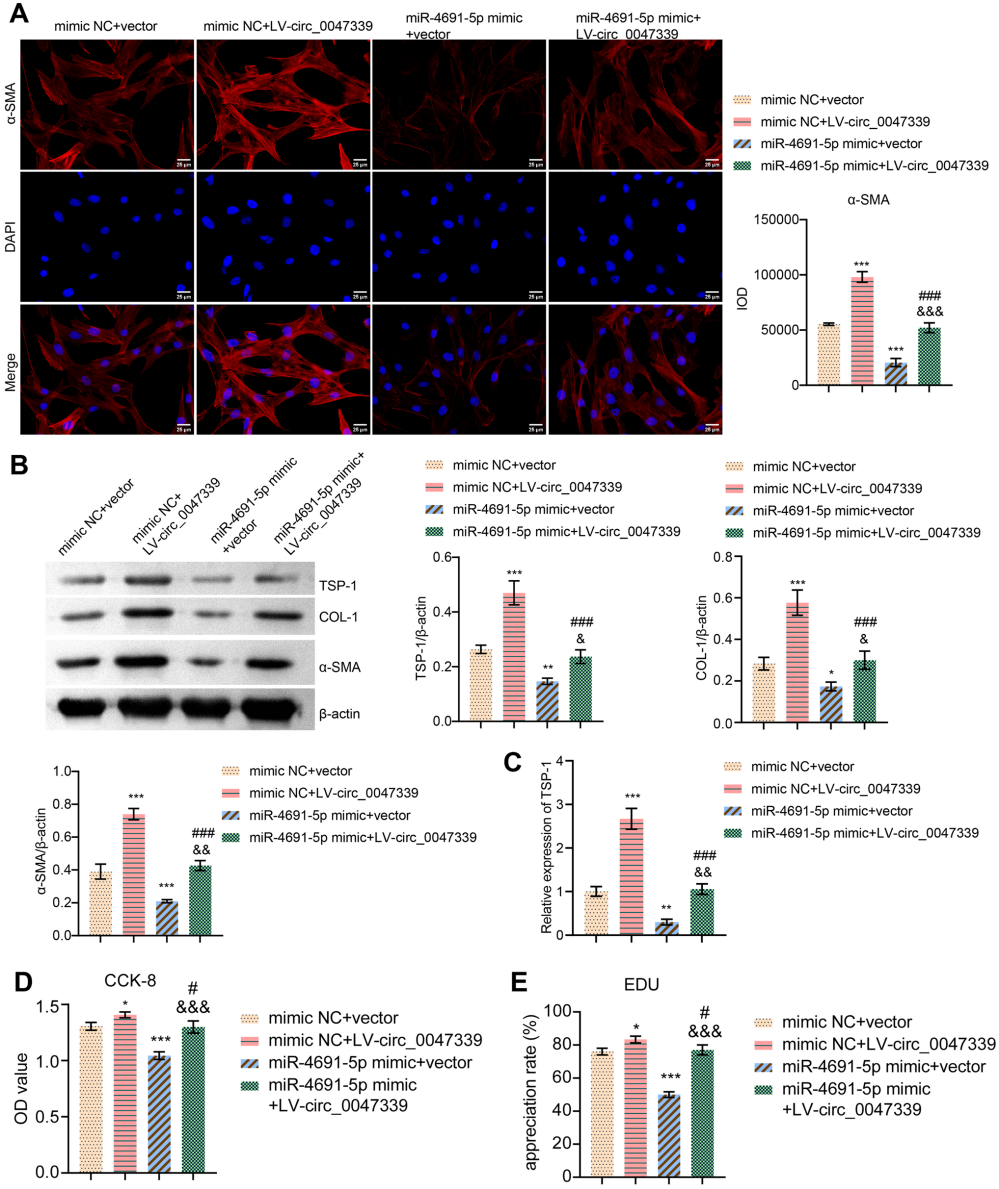

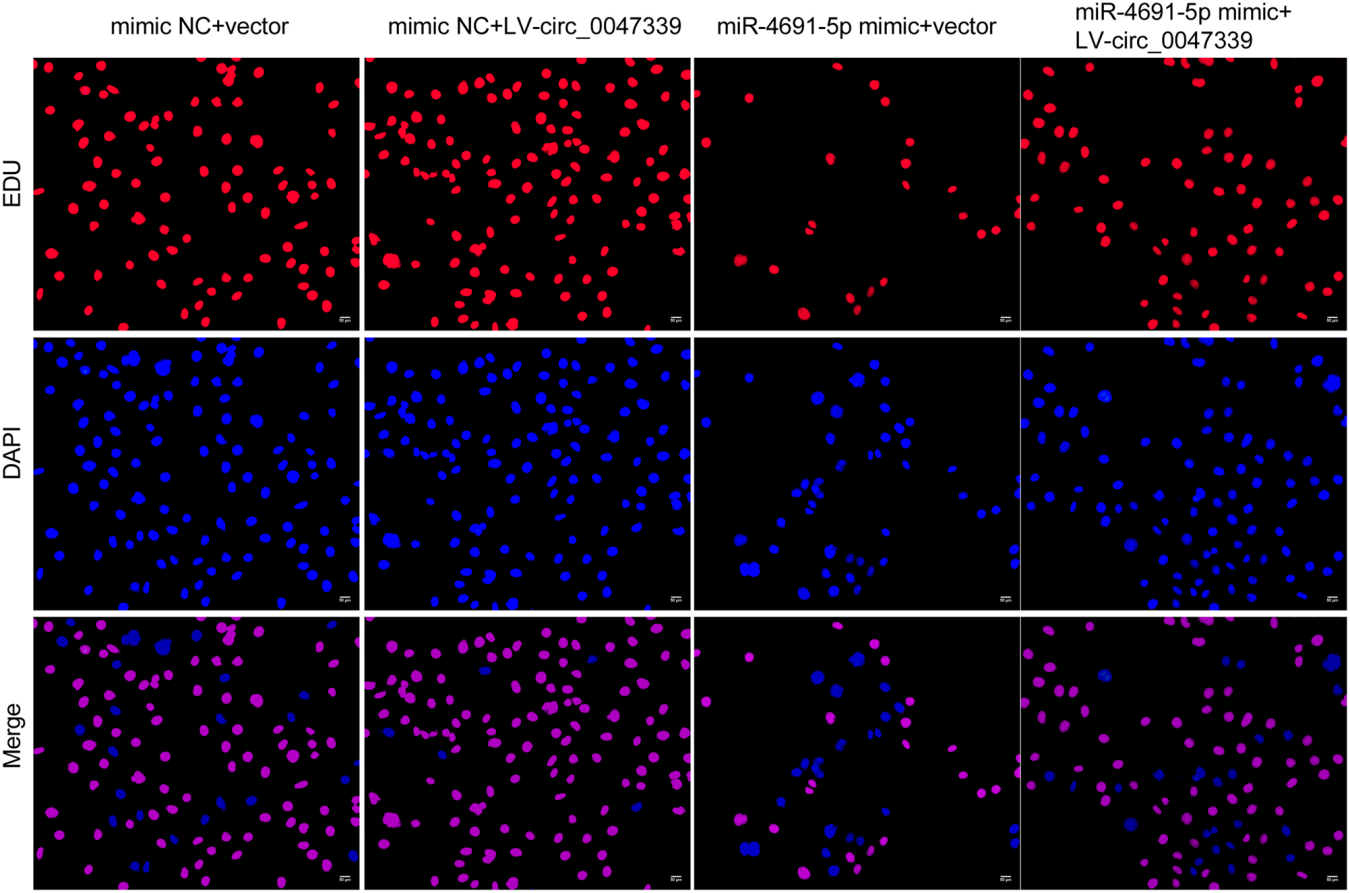
Circ_0047339/miR-4691-5p/TSP-1 interaction network regulated the proliferation, ECM deposition and collagen synthesis of urethral scar fibroblasts. (**A**) The expressions of α-SMA were evaluated by IF, scale bar: 25 μm. (**B**) The protein expression of COL-1, α-SMA and TSP-1 was tested by western blot. (**C**) RT-qPCR was used to assess the expression of TSP-1. (**D**) The cell viability was investigated by CCK-8. (**E**) EDU assay was used to investigate cell proliferation, scale bar: 50 μm. *P < 0.05, **P < 0.01, ***P < 0.001. VS mimic NC + vector group. ^#^P < 0.05, ^##^P < 0.01, ^###^P < 0.001. VS mimic NC + LV-circ_0047339 group. ^&^P < 0.05, ^&&^P < 0.01, ^&&&^P < 0.001. VS miR-4691-5p mimic + vector group.

Because circ_0047339 sponged miR-4691-5p, we subsequently determined the role of miR-4691-5p in vitro. MiR-4691-5p mimic transfection could inhibit the expression of α-SMA and COL-1 protein in urethral scar fibroblasts, while circ_0047339 overexpression could eliminate this inhibition (Fig. 6A,B). These results indicated that circ_0047339/miR-4691-5p interaction regulated ECM deposition and collagen synthesis in urethral scar fibroblasts. As we have proved that TSP-1 is the target of miR-4691-5p, we detected the expression of TSP-1 mRNA and protein by RT-qPCR and western blot. MiR-4691-5p mimic transfection could inhibit the expression of TSP-1 in urethral scar fibroblasts, while circ_0047339 overexpression could eliminate this inhibition (Fig. 6B,C). In addition, we found that miR-4691-5p mimic transfection could inhibit the vitality and proliferation of urethral scar fibroblasts. Overexpression of circ_0047339 could reverse these results (Fig. 6D,E). These results indicated that circ_0047339/miR-4691-5P/TSP-1 network was involved in the regulation of proliferation, ECM deposition and collagen synthesis of urethral scar fibroblasts.

## Discussion

In this study, based on the expression profile analysis of circRNA, we identified circ_0047339 as significantly upregulated circRNA in urethral scar tissue. The loss of function experiment showed that circ_0047339 was related to cell viability, proliferation and fibrosis. Circ_0047339 played its role as a ceRNA that competitively bound miR-4691-5p and then eliminated the endogenous inhibition of miR-4691-5p on the target gene TSP-1. Thus, it could promote the expression of COL-1 and α-SMA and then cell proliferation and fibrosis progress. These results revealed that circ_0047339 promoted the growth and fibrosis of urethral scar fibroblasts through the ceRNA mechanism.

At present, there are few research articles on the relationship between circRNA and urethral stricture. The existing literature mainly focuses on renal fibrosis^20,27^. Other studies have found that circRNA is related to the mechanism of keloid formation^29–31^. Our study used gene chip technology to analyze the differentially expressed circRNA in urethral scar tissue. We found that compared with the normal control group, there were 130 upregulated circRNA and 166 down-regulated circRNA in urethral scar tissue. In addition, KEGG pathway analysis found that differentially expressed circRNA was significantly correlated with ECM–receptor interaction. This opens the door for studying circRNA and urethral stricture, but the relationship between circRNA and the development mechanism of urethral stricture needs further research.

In order to explore how differential circRNA participates in the occurrence and development of urethral stricture, we verified its expression in urethral scar tissue. The results showed that the expression of circ_0047339 in human urethral scar tissue was upregulated, and the difference was the most significant. Therefore, we chose circ_0047339 for the follow-up experiment. Urethral scar formation caused by the overactivation of urethral fibroblasts is the core cytobiological event of urethral stricture^32,33^. We selected urethral fibroblasts to verify the function of circ_0047339. The results showed that circ_0047339 was significantly overexpressed in urethral scar fibroblasts compared with normal urethral fibroblasts. By silencing the expression of circ_0047339, we found that the cell viability, proliferation, and expression of α-SMA and COL-1 of urethral scar fibroblasts decreased. This was consistent with previous results^34^. This evidence suggested that circ_0047339 might participate in urethral stricture by influencing the growth of urethral scar fibroblasts, ECM deposition, and collagen synthesis. It was recommended that circ_0047339 might play an important role in the occurrence and development of urethral stricture.

The research on the biological function mechanism of circRNA has made rapid progress. Among them, circRNA has received the most attention in the function of the miRNA sponge^35,36^. For example, circPTPN12 promotes keloid fibroblasts' growth by activating the Wnt pathway by sponging miR-21-5p^37^. Through dual-luciferase reporter gene detection, we found the interaction of circ_0047339 of miR-4691-5p. MiR-4691-5p can promote the development of liver cancer^38^, but it has not been studied in urethral stricture. Our study was the first to explore the role of miR-4691-5p in urethral stricture. This study found that miR-4691-5p was down-regulated in urethral scar tissue and fibroblasts compared with the control group. Moreover, miR-4691-5p mimic transfection could inhibit the vitality and proliferation of urethral scar fibroblasts. It also could inhibit the expression of α-SMA and COL-1. We speculated that miR-4691-5p might limit fibrogenic signaling in urethral strictures. The dual-luciferase reporter assay showed that the translation activity of TSP-1 3′UTR was significantly inhibited by miR-4691-5p mimic. Correlation analysis showed that miR-4691-5p expression was negatively correlated with the expression of circ_0047339 and TSP-1 in urethral scar tissue. In urethral scar fibroblasts, inhibition of circ_0047339 could up-regulate miR-4691-5p expression and inhibit the expression of TSP-1. These data suggested that circ_0047339 could be used as a molecular sponge of miR-4691-5p to regulate the expression of TSP-1. Inhibition of TSP-1 expression has been proved to inhibit hypertrophic scar development^39,40^. TSP-1 is a regulatory factor that promotes the process of fibrosis, and TGF-β1 can be activated by TSP-1, thus promoting ECM deposition and collagen synthesis^41,42^. Clinical studies have revealed that TGF-β1 levels are significantly up-regulated in patients with urethral strictures^43^. TGF-β1 signaling-mediated hyperactivation of urethral fibroblasts contributes to the progression of traumatic urethral strictures^44^. Targeted intervention in the TSP-1/TGF-β pathway is a therapeutic approach to preventing fibrotic diseases^16^. Our results demonstrated that transfection of miR-4691-5p mimic could inhibit the expression of TSP-1 in urethral scar fibroblasts, thereby inhibiting fibrosis, while overexpression of circ_0047339 could reverse its inhibitory effect. These results suggested that circ_0047339/miR-4691-5p/TSP-1 network participates in the growth and activation of urethral scar fibroblasts.

It is important to emphasize some limitations related to this study. First, the role of only circ_0047339 was verified, while the expression profile of other circRNAs in urethral stricture remains to be explored. In addition, circ_0047339 was highly expressed in urethral scar tissue, but the reason or mechanism for its high expression needs to be further studied through systematic experimental analysis. Finally, TSP-1 is an extracellular matrix protein that mediates cell–matrix and cell–cell interactions^14^. Many studies have demonstrated that TSP-1 is the main regulator of TGF-β activation and can regulate the expression of TGF-β/Col-1/α-SMA^16,41,45^. Herein, we found that circ_0047339 promoted the expression of TSP-1 by sponging miR-4691-5p in urethral stricture. Meanwhile, down-regulation of circ_0047339 inhibited the expression of Col-1/α-SMA. Therefore, we speculated that circ_0047339 might affect Col-1/α-SMA by affecting the miR-4691-5p/TSP-1 axis. However, the mechanism of TSP-1’s effect on Col-1/α-SMA is quite complex. More experimental data will be needed to prove its specific regulatory mechanism. This is the limitation of our work. We will conduct cell and animal experiments to analyze this in-depth in future research.

## Conclusion

Our research showed that circRNA could regulate the development of urethral stricture. Circ_0047339 was upregulated in human urethral scar tissue and urethral scar fibroblasts. Circ_0047339 silence could inhibit the proliferation of urethral scar fibroblasts and the expression of α-SMA and COL-1. Circ_0047339 regulated fibroblast proliferation, ECM deposition and collagen synthesis by increasing TSP-1 expression as an endogenous miR-4691-5p sponge.

## Supplementary Information

Supplementary Figure S1.

Supplementary Figure S2.

Supplementary Figure S3.

Supplementary Figure S4.

## Data Availability

All data included in this study are available upon request by contact with the first author or corresponding author. The datasets generated and/or analysed during the current study are available in the [GEO] repository, [https://www.ncbi.nlm.nih.gov/gds/?term], and the accession number is GSE209937.
