# Supplementary figures and images for: Circ_0047339 promotes the activation of fibroblasts and affects the development of urethral stricture by targeting the miR-4691-5p/TSP-1 axis
Source: Sci Rep. 2022 Aug 30;12:14746. doi: 10.1038/s41598-022-19141-4 (PMC9428161; doi:10.1038/s41598-022-19141-4)

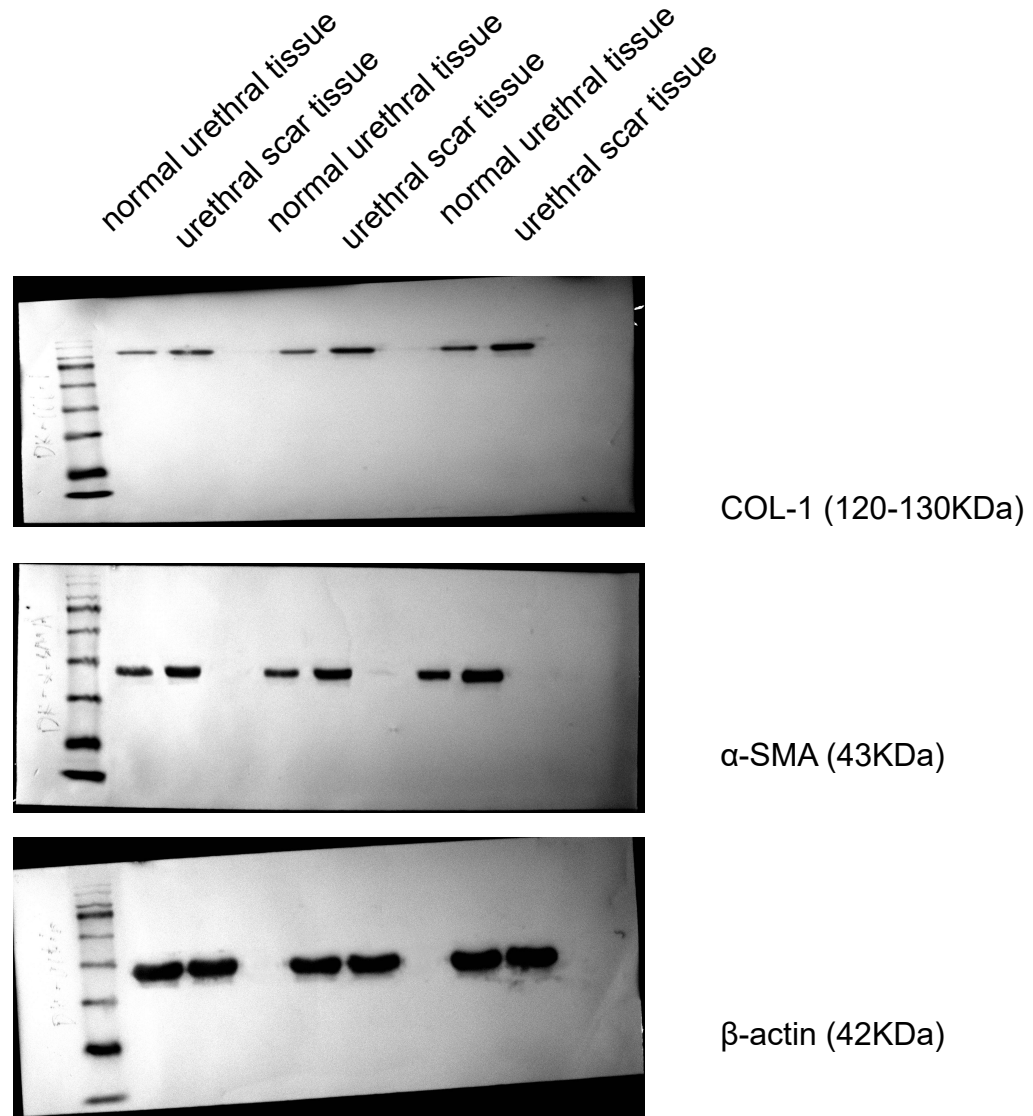

**Figure S1 Un-cropped image of Figure 3A**

Supplement: Supplementary file 1 — Supplementary Figure S1. [file 41598_2022_19141_MOESM1_ESM.pdf]

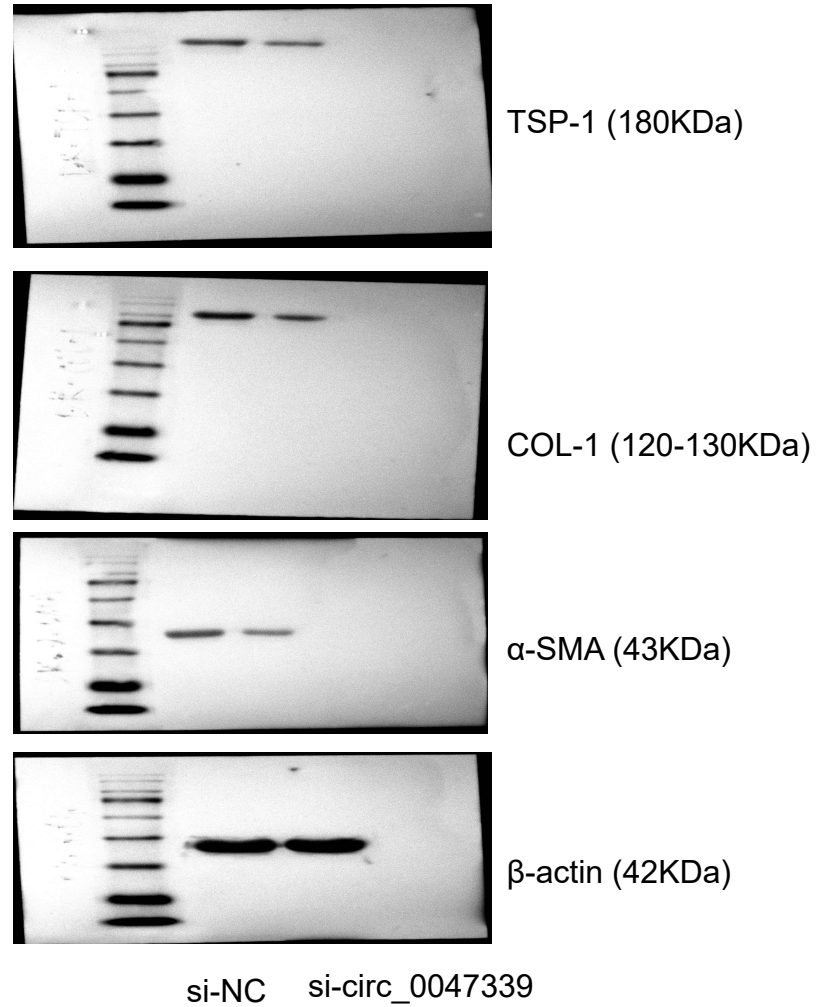

**Figure S3 Un-cropped image of Figure 4E**

Supplement: Supplementary file 3 — Supplementary Figure S3. [file 41598_2022_19141_MOESM3_ESM.pdf]

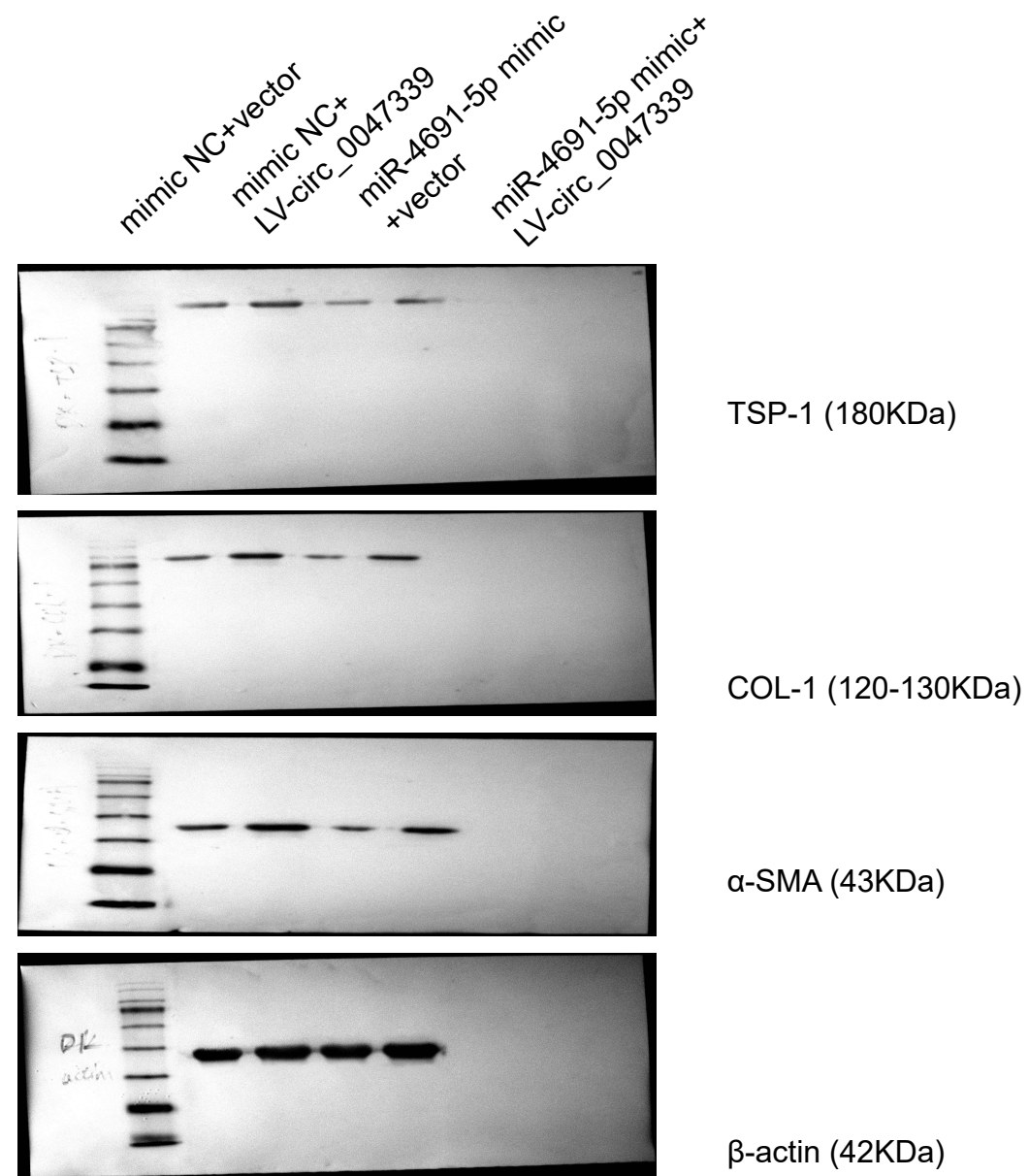

**Figure S4 Un-cropped image of Figure 6B**

Supplement: Supplementary file 4 — Supplementary Figure S4. [file 41598_2022_19141_MOESM4_ESM.pdf]
