# Supplementary material for: Circ_0047339 promotes the activation of fibroblasts and affects the development of urethral stricture by targeting the miR-4691-5p/TSP-1 axis
Source: Sci Rep. 2022 Aug 30;12:14746. doi: 10.1038/s41598-022-19141-4 (PMC9428161; doi:10.1038/s41598-022-19141-4)
Supplement: Supplementary file 2 — Supplementary Figure S2. [file 41598_2022_19141_MOESM2_ESM.pdf]

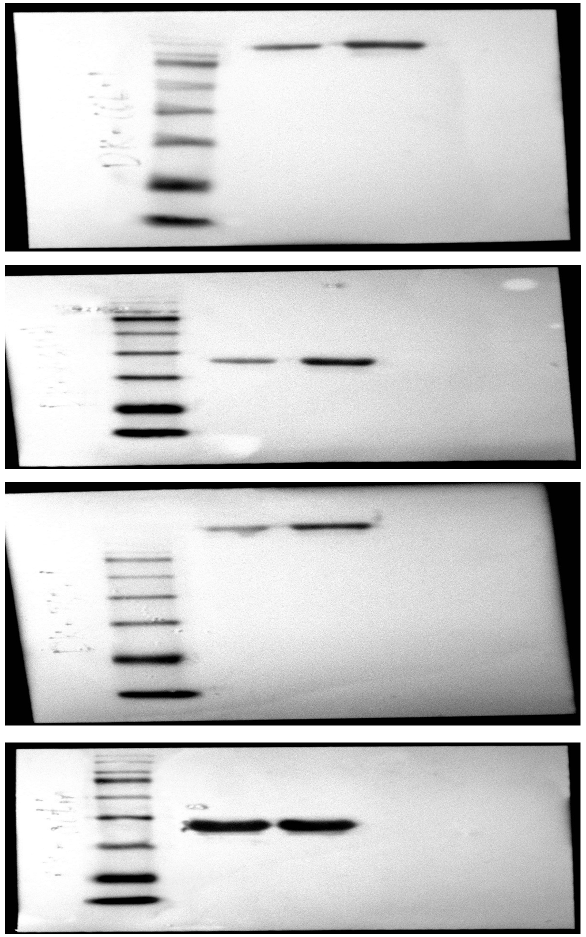


**Figure S2 Un-cropped image of Figure 4B**
